# Protective Effects of Positive Lysosomal Modulation in Alzheimer's Disease Transgenic Mouse Models

**DOI:** 10.1371/journal.pone.0020501

**Published:** 2011-06-10

**Authors:** David Butler, Jeannie Hwang, Candice Estick, Akiko Nishiyama, Saranya Santhosh Kumar, Clive Baveghems, Hollie B. Young-Oxendine, Meagan L. Wisniewski, Ana Charalambides, Ben A. Bahr

**Affiliations:** 1 Neurosciences Program, University of Connecticut, Storrs, Connecticut, United States of America; 2 Department of Pharmaceutical Sciences, University of Connecticut, Storrs, Connecticut, United States of America; 3 William C. Friday Laboratory, Biotechnology Research and Training Center, University of North Carolina Pembroke, Pembroke, North Carolina, United States of America; 4 Department of Physiology and Neurobiology, University of Connecticut, Storrs, Connecticut, United States of America; 5 Department of Molecular and Cell Biology, University of Connecticut, Storrs, Connecticut, United States of America; Boston University, United States of America

## Abstract

Alzheimer's disease (AD) is an age-related neurodegenerative pathology in which defects in proteolytic clearance of amyloid β peptide (Aβ) likely contribute to the progressive nature of the disorder. Lysosomal proteases of the cathepsin family exhibit up-regulation in response to accumulating proteins including Aβ_1–42_. Here, the lysosomal modulator Z-Phe-Ala-diazomethylketone (PADK) was used to test whether proteolytic activity can be enhanced to reduce the accumulation events in AD mouse models expressing different levels of Aβ pathology. Systemic PADK injections in APP_SwInd_ and APPswe/PS1ΔE9 mice caused 3- to 8-fold increases in cathepsin B protein levels and 3- to 10-fold increases in the enzyme's activity in lysosomal fractions, while neprilysin and insulin-degrading enzyme remained unchanged. Biochemical analyses indicated the modulation predominantly targeted the active mature forms of cathepsin B and markedly changed Rab proteins but not LAMP1, suggesting the involvement of enhanced trafficking. The modulated lysosomal system led to reductions in both Aβ immunostaining as well as Aβ_x-42_ sandwich ELISA measures in APP_SwInd_ mice of 10–11 months. More extensive Aβ deposition in 20-22-month APPswe/PS1*Δ*E9 mice was also reduced by PADK. Selective ELISAs found that a corresponding production of the less pathogenic Aβ_1–38_ occurs as Aβ_1–42_ levels decrease in the mouse models, indicating that PADK treatment leads to Aβ truncation. Associated with Aβ clearance was the elimination of behavioral and synaptic protein deficits evident in the two transgenic models. These findings indicate that pharmacologically-controlled lysosomal modulation reduces Aβ_1–42_ accumulation, possibly through intracellular truncation that also influences extracellular deposition, and in turn offsets the defects in synaptic composition and cognitive functions. The selective modulation promotes clearance at different levels of Aβ pathology and provides proof-of-principle for small molecule therapeutic development for AD and possibly other protein accumulation disorders.

## Introduction

Alzheimer's disease (AD) is the most prevalent form of senile dementia, and is characterized by progressive compromise of synaptic integrity and cognitive functions. Aβ accumulation in the brain is a hallmark of AD pathology, and Aβ_1–42_ species have been implicated in the disruption of synaptic function and neuronal loss [1]. In addition to extracellular deposition, accumulation of Aβ also occurs intraneuronally [2]–[8], likely due to defective clearance and in many cases occurring prior to amyloid plaque formation. The clearance rates for Aβ peptides were indeed found to be slower in AD patients as compared to the rates in cognitively normal individuals [9]. One conclusion is an imbalance in Aβ production vs. clearance that implicates a plausible mechanism for the Aβ dysregulation in the more common late-onset AD, and perhaps a contributing factor in familial AD.

Intraneuronal Aβ_1–42_ is found in the brains of Alzheimer patients and individuals with mild cognitive impairment [2], [10], thus there is growing evidence that such intracellular accumulation is an early indicator of neuronal compromise that correlates with cognitive decline. Synaptic dysfunction and deterioration are exhibited by Aβ–containing neurons and the intraneuronal accumulation is associated with cognitive deficits in animal models [2], [4], [6]–[8], [11]. It is widely held that reducing protein accumulation events is critical for slowing the progression of AD, especially those produced by the aggregation-prone Aβ_1–42_ peptide. Potential targets include Aβ-degrading enzymes such as neprilysin, insulin-degrading enzyme, and endothelin-converting enzyme, and these proteases may in fact be responsible for Aβ homeostasis in the brain [12]–[15]. The lysosomal hydrolase cathepsin B has also been found to cleave Aβ_1–42_ into less amyloidogenic species [16]. This is of particular interest since extracellular Aβ_1–42_ can be taken up by neurons in AD-vulnerable subfields and sequestered into lysosomes [11], [17], [18], thus lysosomal cathepsin activity may be important for the clearance of the peptide.

The endosomal/lysosomal system plays an important role in protein clearance, and its enhancement has been suggested as a strategy to reduce aberrant protein accumulation in age-related neurodegenerative disorders [19]–[24]. Interestingly, the endosomal/lysosomal system exhibits evidence of regulatory events in response to accumulating proteins found in AD [16], [24]–[27] and areas of the aged brain [20], [28], [29]. The cathepsin family of lysosomal hydrolases appears to be particularly responsive to AD-related proteins accumulating in neurons. Protein accumulation stress, including that produced by Aβ_1–42_, markedly up-regulates the message, protein, and activity levels of the cysteine protease cathepsin B (EC 3.4.22.1), the aspartyl protease cathepsin D (EC 3.4.23.5), and other isoforms [16], [19], [20], [24]. These protease responses may be reflective of early compensatory processes that keep protein accumulation events partially in check and account for the gradual nature of AD pathology. The response by cathepsin B in APP_SwInd_ mice failed to occur in older animals [16], suggesting that reduced efficiency of this compensatory pathway contributes to the age-related vulnerability of the brain.

To investigate the effects of lysosomal enhancement on protein accumulation pathology, a positive modulator of the lysosomal system was tested in transgenic mouse models of AD for its ability to promote cathepsin activity and protein clearance. The modulator selectively enhanced cathepsin B levels in the CNS, resulting in reduced Aβ_1–42_ levels and increased measures of a truncated Aβ_1–38_ peptide. Associated with the enhanced clearance of intra- and extracellular Aβ was the corresponding protection of synaptic integrity and improved cognitive ability. These findings provide further evidence that lysosomal enzymes can regulate the level of Aβ in the brain, and they indicate a minimally invasive approach to enhance lysosomal degradation of Aβ as a treatment for AD.

## Materials and Methods

### Animals and Injection Schedule

All transgenic mice and non-transgenic littermates were obtained from Jackson Laboratories (Bar Harbor, ME) and housed in vivarium facilities until the desired age. The APPswe/PS1*Δ*E9 mice, strain B6C3-Tg(APPswe,PSEN1dE9)85Dbo/J (APP-PS1; stock number 004462) were used at 20–22 months of age. APP_SwInd_ mice received from Jackson laboratories (stock number 004661) expressed only 15% of the transgene copy number normally expressed by the B6.Cg-Tg(PDGFB-APPSwInd)20 Lms/2J strain. The mice exhibited lower levels of Aβ deposits compared to the original APP_SwInd_ J20 line [30], and were used at 10–11 months as a model of early Aβ pathology. Genotype was confirmed by PCR on tail DNAs. Non-transgenic Sprague-Dawley rats (Charles River Laboratories, Wilmington, MA) were used at 11–12 days postnatal to prepare hippocampal slice cultures, following a routine protocol with Millicell-CM inserts (Millipore, Bedford, MA) [19]–[21]. All studies were carried out in strict accordance with the recommendations from the Guide for the Care and Use of Laboratory Animals of the National Institutes of Health. Animal use and analyses were conducted in accordance with approved protocols from the Animal Care and Use Committees of the University of Connecticut (Protocol A09-008) and the University of North Carolina–Pembroke (Protocols 2009-001 and 2010-003). Mice were handled daily for >1 week and subsequently received daily i.p. injections of 18–20 mg/kg Z-Phe-Ala-diazomethylketone (PADK), obtained from Bachem Americas, Inc. (N-1040; Torrance, CA). PADK solutions were initially prepared at 24 mg/ml in dry DMSO, and slowly diluted with PBS to 12 mg/ml. Control mice were injected with the corresponding amount of vehicle (50% PBS and 50% DMSO). Consistent lysosomal modulation results were obtained from at least three PADK preparations represented by different lot numbers.

### Behavioral Paradigms

Mice were handled and familiarized with the T-maze and suspended bar setup prior to the start of PADK injections. A day before the end of the injection schedule, spontaneous alternation behavior was assessed to measure episodic memory deficits in APP-PS1 mice. Transgenic and control mice were placed at the intersection of a T-maze, and entries across each arm's threshold were observed with a closed-circuit monitor for a 10-min period. A minimum of 15 entries was used to determine percent alternations when compared to total alternations possible. An alternation was a succession of entries into 3 different arms of the maze. Mobility in the T-maze or in a novel open field was assessed by grid crossings in the first 3 min of exploration. APP_SwInd_ mice and their respective controls were assessed on the suspended rod test, in which the mice were placed in the middle of a 1-cm diameter rod suspended 40-cm over a padded service with platforms in sight 56 cm apart. Time of maintained uprightness was recorded on the third trial as the mice attempted to reach a platform during a period of 60 sec. They were also tested in an open field of 55 cm×36 cm to determine exploratory distance during novel exposure to the environment and during subsequent re-exposure 24 h later. Grid crossings were assessed for 5 min using a closed-circuit camera.

### Tissue Preparation

Immediately following behavior testing, brain tissue was removed and prepared for analyses. Some animals were anesthetized and perfused with 4% paraformaldehyde prior to dissecting brains for tissue sectioning and subsequent hematoxylin-eosin staining or immunofluorescence protocols. For the remaining mice, brains were removed and quickly dissected in ice-cold buffer containing 0.32 M sucrose, 5 mM HEPES (pH 7.4), 1 mM EDTA, 1 mM EGTA, and the protease inhibitors aprotinin, leupeptin, bestatin, E-64, pepstatin A (each at 2 µg/ml), and 4-(2-aminoethyl)benzenesulfonyl fluoride (0.3 mM) (unless otherwise stated, reagents were from Sigma-Aldrich Co., St. Louis, MO). Regions were separated from one hemi-forebrain and snap-frozen in liquid nitrogen for later homogenization in lysis buffer containing protease inhibitors. The other hemi-forebrain was either 1) fixed in phosphate-buffered 4% paraformaldehyde for immunocytochemistry, 2) mechanically homogenized in appropriate buffer conditions to collect the extracellular-enriched fraction following centrifugation [31], or 3) homogenized in ice-cold 0.3 M sucrose with 1 mM EDTA for the isolation of lysosomes (see below).

### Immunoblotting and ELISA

Equal amounts of sample protein were separated on standard or tris-tricine gradient gels, and transferred to nitrocellulose for antibody staining. Antibodies utilized were developed against cathepsin B (1∶200; Millipore, Bedford, MA), cathepsin D (1∶300; Cortex Biochemicals, San Leandro, CA), neprilysin (1∶300; Millipore), insulin-degrading enzyme (1∶200; Covance, Princeton, NJ), α-secretase (1∶500, against amino acids 732–748 of human TNF-α converting enzyme; ProSci, Poway, CA), actin (1∶1,000; Sigma-Aldrich Co.), amino acids 1–16 of human Aβ (6E10, 1∶500; Covance), human sAPPα (2B3, 1∶100; IBL International, Hamburg, Germany), human sAPPβ-sw (6A1, 1∶200; IBL), Rab5a (1∶200; Santa Cruz Biotechnology, Santa Cruz, CA), Rab7 (1∶100; Santa Cruz Biotechnology), LAMP1 (1∶200; GeneTex Inc., Irvine, CA), synapsin II (1∶200; Millipore), synaptophysin (Chemicon, Temecula, CA), and the carboxy termini of AMPA receptor subunit GluA1 [19]. Secondary antibodies were from Bio-Rad (Richmond, CA) and immunoreactive bands were assessed for integrated optical density with BIOQUANT software (Nashville, TN). Equal aliquots of soluble homogenate fractions were also assessed by ELISA protocols for the specific detection of Aβ_x-42_ and Aβ_x-38_ species using antibodies (12F4 and BA1–13, respectively) and reagents from Covance. Chemiluminescence detection was measured with a SpectraMax L Luminescence Reader (Molecular Devices, Sunnyvale, CA) and converted to femtomoles per milligram sample protein using standard curves generated with pure Aβ_1-42_ and Aβ_1-38_ peptides (Bachem).

### Immunocytochemistry

Fixed tissue was cryoprotected and serial sectioned at 20-µm thickness. Immunolabeling followed standard free-floating methods using anti-cathepsin B (Millipore), anti-NeuN (Invitrogen, Carlsbad, CA), 6E10 antibody (Covance), anti-LAMP1 (BD Pharmingen, San Jose, CA), and monoclonal antibody that selectively labels Aβ_1–42_ (Covance). Immunofluorescence used appropriate secondary antibodies from Invitrogen, and images were captured with a Zeiss fluorescence microscope system. For quantitative analysis of integrated fluorescence intensity, images from the different treatment groups received the same gain, exposure time, intensity threshold, and other measurement parameters that were capsulated within each image file. Avidin-biotin-peroxidase protocols used Vectastain kits (Vector Laboratories, Burlingame, CA) with 3,3′-diaminobenzidine as the chromogen, and images were acquired with a digital microscope system (Olympus AX70). Densitometric quantification was conducted with a BIOQUANT Image Analysis System. Treatment groups were immunostained together and analyzed under the same instrument settings. Equally spaced coronal sections along the rostral-caudal axis of the hippocampus were used to determine the average immunoreactivity intensity across four different cortical view-fields for each mouse. To assess hippocampal subfields, the CA1 field was investigated with view-fields of CA1b and the initial area of CA1c. For CA3, view-fields were selected from the zone immediately before the dentate gyrus envelope. In the dentate gyrus, granule cells of the stratum granulosum were counted in view-fields from the middle segment of the inner blade. In addition to integrated fluorescence and colormetric intensity measures, extracellular deposition was assessed with an autothreshold function and the area of immunostaining was expressed as percentage of the total view-field area being evaluated.

### β-Secretase Activity Assay

Compounds were tested against the activity of β-secretase (β-site APP cleaving enzyme-1; BACE1) using Sigma's SensiZyme BACE1 Activity Assay Kit. The kit uses a constructed procaspase-3 variant containing the modified cleavage sequence Gly-Ser-Ser-Glu-Ile-Ser-Tyr-Glu-Val-Glu-Phe-Arg-Glu-Phe which is cleaved by β-secretase after the Tyr-Glu residues [32]. Increasing concentrations of potential inhibitors, including β-secretase inhibitor IV (EMD Chemicals, Gibbstown, NJ), were incubated with 10 ng/ml of C-terminal FLAG-tagged recombinant human β-secretase from HEK 293 cells. Activity was expressed as absorbance units produced by the caspase-3 colorimetric substrate N-acetyl-Asp-Glu-Val-Asp-*p*-nitroanilide and measured with a SpectraMax M3 Microplate Reader.

### Lysosome Isolation and Cathepsin Activity

Dissected hemi-brains were quickly separated into regions and each immediately homogenized in ice-cold 0.3 M sucrose with 1 mM EDTA and centrifuged at 750×g for 10 min. The collected supernatant was incubated with 2 mM CaCl_2_ at 37°C for 5 min then layered over 24% Percoll in 0.32 M sucrose containing 1 mM EDTA (pH 5.5). After centrifugation at 20,000×g for 18 min the gradients were fractionated, and those determined to be negative (upper four fractions) or positive (lower two) for cathepsin B by immunoblot were diluted with 5 volumes of sucrose solution to separate organelles from the Percoll by centrifuging at 40,000×g for 60 min. The pellets were resuspended and protein content determined with the Pierce BCA assay (Thermo Scientific, Rockford, IL). The resulting fractions were aliquoted, lysed in 18 mM citrate, and assessed for cathepsin B proteolytic activity using the Z-Arg-Arg AMC substrate, the fluorogenic Calbiochem assay kit (EMD Chemicals), and the Molecular Devices SpectraMax M3 Microplate Reader. Potential inhibitors were also tested in the cathepsin B activity assay, using equal aliquots of Triton X-100 solubilized brain homogenate pre-treated for 30 min with increasing concentrations of the agents.

### Statistical and Data Analyses

Means of measures from image analysis of immunoblots and tissue staining, ELISA tests, behavioral assessment, enzyme activity, and other experiments were evaluated with unpaired t-tests, unpaired Mann-Whitney U-tests, or across >2 treatment groups with analyses of variance (ANOVA) followed by the Tukey's multiple comparison post hoc tests using Prism software (GraphPad, San Diego, CA). Nonlinear regression was used to fit enzyme activity inhibition data to one-site competitive binding equations. Linear regression analyses tested for significant correlations between Aβ species and cathepsin B enhancement or between GluA1 immunoreactivity and the lysosomal enhancement.

## Results

**Figure 1:**
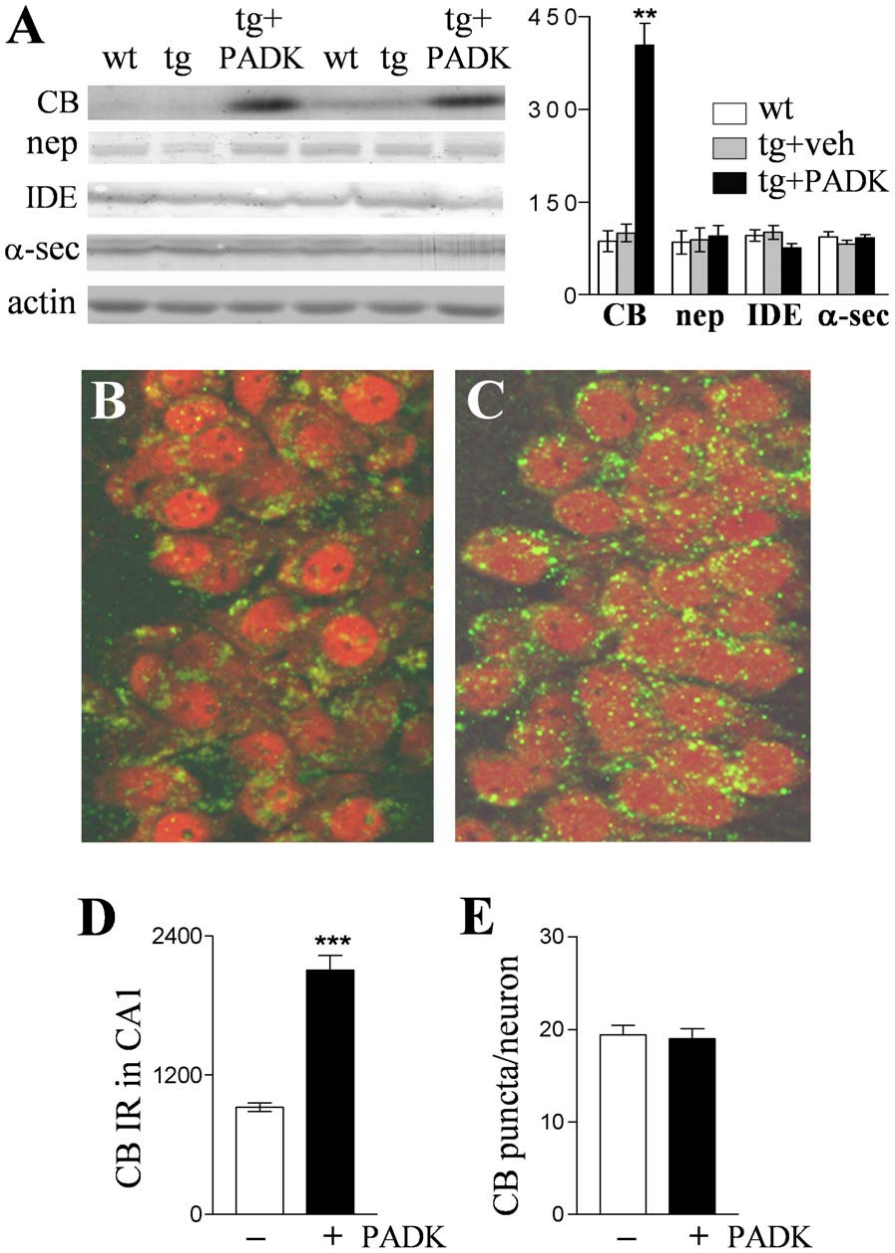
The lysosomal modulator PADK selectively enhances cathepsin B levels in APP_SwInd_ mice. The 10–11-month transgenic mice (tg) were injected i.p. daily with either PADK (20 mg/kg; n = 13) or vehicle (veh; n = 10) for 9 days. Brain homogenates from the transgenic mice and from vehicle-treated wildtypes (wt; n = 13) were analyzed by immunoblot for the active form of cathepsin B (CB), neprilysin (nep), insulin-degrading enzyme (IDE), α-secretase (α-sec), and actin (A). Mean immunoreactivities±SEM were determined by image analysis and plotted. Hippocampal photomicrographs from vehicle- (B) and PADK-treated mice (C) show cathepsin B immunostaining (green) in pyramidal neurons counterstained with anti-NeuN (red); view-field width is 75 µm. The CA1 zone was assessed for cathepsin B immunoreactivity (mean intensity±SEM) in the pyramidal layer (D) and for the number of cathepsin B-positive puncta per neuron (E). Tukey post hoc test: **P<0.001; unpaired t-test: ***P<0.0001.

To elicit lysosomal enhancement in mouse models of AD, we administered Z-Phe-Ala-diazomethylketone (PADK), a weak inhibitor of cathepsin B and L (cathepsin B IC_50_ = 9.4±2.4 µM) as well as a lysosomal modulator previously shown to cause a feedback response involving marked up-regulation of cathepsin isoform expression *in vitro* [19]–[21]. The lysosomal modulator (20 mg/kg) was injected i.p. daily for 9 days into 10–11-month APP_SwInd_ mice which express the human APP gene with the Swedish (K670N/M671L) and Indiana (V717F) mutations [30], resulting in a marked increase in the active isoform of cathepsin B in the brain as compared to vehicle-injected transgenic mice (Fig. 1A; ANOVA *P*<0.0001, post hoc test *P*<0.001; n = 13). Cathepsin B immunoreactivity levels were enhanced >4 fold in hippocampal samples, and 3-fold or greater increases were found in samples from neocortex, frontal cortex, and mesencephalon (Table 1). Measures of Aβ-degrading proteases neprilysin and insulin-degrading enzyme, as well as α-secretase which prevents Aβ production, were not altered (Fig. 1A and Table 2), thus the PADK-mediated lysosomal modulation was produced in a selective manner. Similar selectivity was also evident for the PADK effect in 20–22-month APPswe/PS1ΔE9 mice (APP-PS1; Table 2), which express a chimeric mouse/human APP and human presenilin 1 directed to CNS neurons [33]. Significant cathepsin B up-regulation was found in different brain regions of the APP-PS1 mice, with hippocampus exhibiting the largest increase of >8 fold (Table 1).

**Table 1:** PADK-mediated enhancement across brain regions of transgenic mouse models.

Brain Region	APP_SwInd_	APP-PS1
neocortex	3.0±0.17	7.3±0.60
frontal cortex	3.2±0.33	6.8±1.3
hippocampus	4.3±0.38	8.4±0.85
mesencephalon	3.7±0.70	4.9±0.21

APP_SwInd_ (10–11 months of age) and APPswe/PS1ΔE9 mice (APP-PS1; 20–22 months) were injected i.p. daily with PADK (20 mg/kg; n = 11−13) or vehicle (n = 10) for 9–11 days. Active cathepsin B in tissue homogenates was measured by immunoblot, and the mean levels were compared to the respective mean immunoreactivity in vehicle-injected transgenic samples to determine the fold increase across brain regions (±SEM).

**Table 2:** PADK selectively enhances cathepsin B levels in two transgenic mouse models.

	APP_SwInd_+veh	APP_SwInd_+PADK	APP−PS1+veh	APP−PS1+PADK
CB	95.3±14.2	404.6±36.0***	44.5±15.2	376.0±38.6***
nep	89.1±19.2	95.3±16.6	43.0±6.5	31.5±6.6
IDE	101.1±11.0	76.4±6.5	96.1±8.3	89.5±6.0
α-sec	82.3±6.0	92.0±5.2	60.1±8.9	58.9±6.2
LAMP1	98.4±5.4	102.2±8.6	46.1±9.9	45.8±4.6

APP_SwInd_ and APP-PS1 mice were injected i.p. daily with PADK (20 mg/kg; n = 11−13) or vehicle (n = 10) for 9–11 days. Hippocampal homogenates were analyzed by immunoblot and mean immunoreactivities are shown for active cathepsin B (CB), neprilysin (nep), insulin-degrading enzyme (IDE), α-secretase (α-sec), and LAMP1.

***P<0.0001, unpaired t-test.

**Figure 2:**
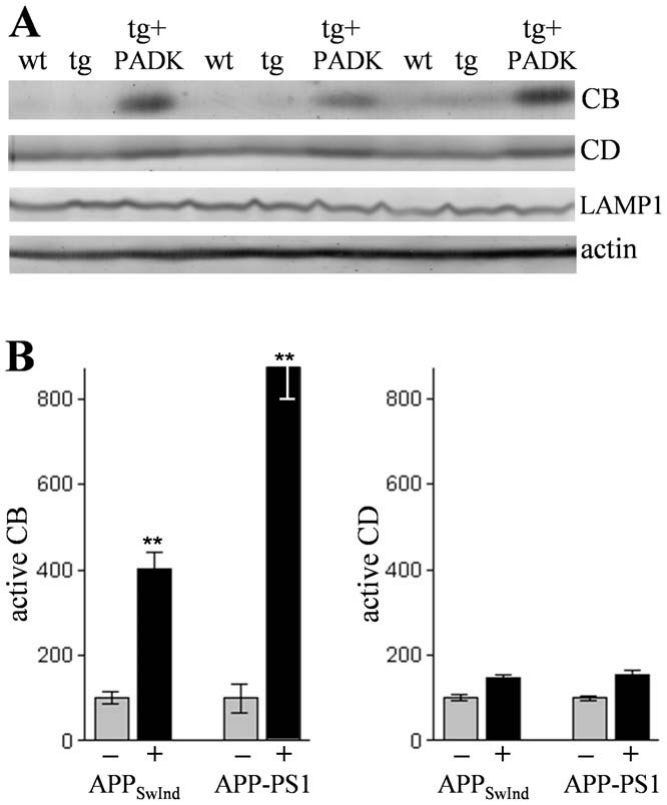
PADK modulates cathepsin B more than cathepsin D in APP_SwInd_ mice. The APP_SwInd_ transgenic mice (tg) were subjected to 9 daily injections of PADK (20 mg/kg; n = 13) or vehicle (n = 10), and wildtype mice (wt; n = 13) were injected with vehicle. Hippocampal homogenate samples were analyzed by immunoblot for the active isoform of cathepsin B (CB), the 33-kDa mature cathepsin D (CD), the lysosomal marker LAMP1, and actin (A). Mean immunoreactivities±SEM for CB and CD were determined and the respective data normalized with vehicle-treated transgenic groups (–) set at 100% (B). ANOVA across the 8 normalized groups: *P*<0.0001; Tukey post hoc tests: **P<0.001.

In immunocytochemistry images, intracellular cathepsin B was revealed as punctate staining (green) characteristic of lysosomal organelles in hippocampal CA1 pyramidal neurons (Fig. 1B), and the intensity of such immunostaining was enhanced after PADK treatment (Fig. 1C). Neurons were counterstained with anti-NeuN (red), and PADK elicited no apparent change in neuronal density or morphology. Quantitative analysis of the fluorescence intensity across the stratum pyramidale confirmed an increase in cathepsin B immunoreactivity (*P*<0.0001; Fig. 1D). On the other hand, the number of cathepsin B-positive organelles per pyramidal neuron (n = 62) was found to be unchanged in the view-fields (Fig. 1E), and the lysosome-associated membrane glycoprotein LAMP1 was also unaltered in blot samples from the different treatment groups (Fig. 2A, Table 2). ANOVA assessment of two different cathepsins (B and D) across all transgenic treatment groups found that only cathepsin B increased with PADK treatment (Fig. 2). Thus, the 2- to 3-fold increase in intracellular cathepsin B staining, in the absence of a change in lysosome number, appears to represent the primary PADK effect in neurons. Note that a one-way nonparametric Kruskal-Wallis analysis followed by Dunn's post test of the 33-kDa cathepsin D form alone revealed significant albeit small increases produced by PADK in the APP_SwInd_ and APP-PS1 samples (*P*<0.05).

**Figure 3:**
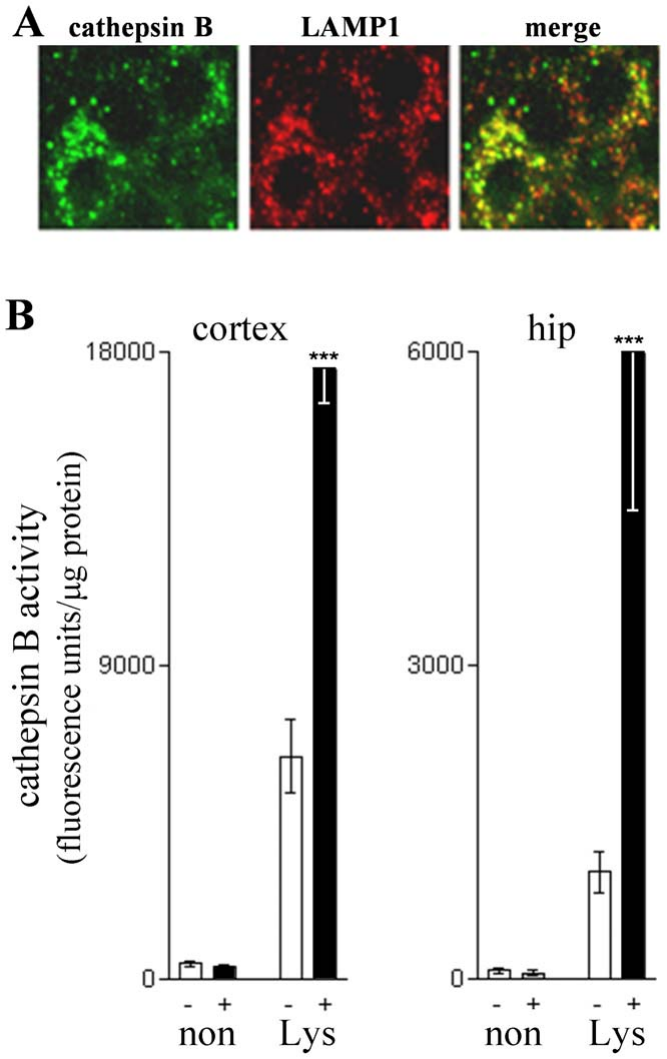
PADK-modulated cathepsin B is localized to lysosomes. From PADK-treated mice (20 mg/kg/day×9 days) exhibiting increased levels of active cathepsin B, fixed hemi-brains were sectioned and double-stained for cathepsin B (green) and the lysosomal marker LAMP1 (red). Individual antigen labeling and the merged image from hippocampal CA1 show that PADK-modulated cathepsin B highly co-localizes with LAMP1-positive organelles in pyramidal neurons. View-field width: 35 µm. To localize cathepsin B activity, APP_SwInd_ mice were injected daily with vehicle (–) or 18 mg/kg PADK (+) for 10 days, and cortical and hippocampal regions were subsequently dissected to isolate lysosomes. Lysosomal fractions (Lys) and non-lysosomal fractions (non) were separated using Percoll gradients, and the two types of fractions were separately pooled and assessed for protein content and hydrolase activity with Z-Arg-Arg AMC (mean specific activity plotted±SEM). Unpaired Mann-Whitney U-test compared to lysosomal fractions from vehicle-treated mice: ***P<0.0001.

To further test whether the intracellular modulation is actually influencing lysosomal cathepsin B, localization of the PADK effect was evaluated in brain tissue double-stained for cathepsin B and LAMP1 (Fig. 3A). As evident in the merged immunofluorescence image, the PADK-modulated cathepsin B co-localized with LAMP1-positive organelles in CA1 pyramidal neurons. Together, the findings indicate that the lysosomal modulator enhances cathepsin B content in lysosomes. APP_SwInd_ mice injected with vehicle or 18 mg/kg PADK daily for 10 days were also assessed for cathepsin B activity in isolated lysosomes using the Z-Arg-Arg AMC probe (Fig. 3B). Rapidly dissected brain regions were subjected to subcellular fractionation in Percoll gradients, and equal protein aliquots from the lysosomal and non-lysosomal fractions were evaluated for hydrolase activity that was blocked by the cathepsin B inhibitor CA074me. Note that in the different brain regions tested, only the lysosomal fractions exhibited PADK-dependent increases in cathepsin B activity of 3-10 fold (*P*<0.0001).

**Figure 4:**
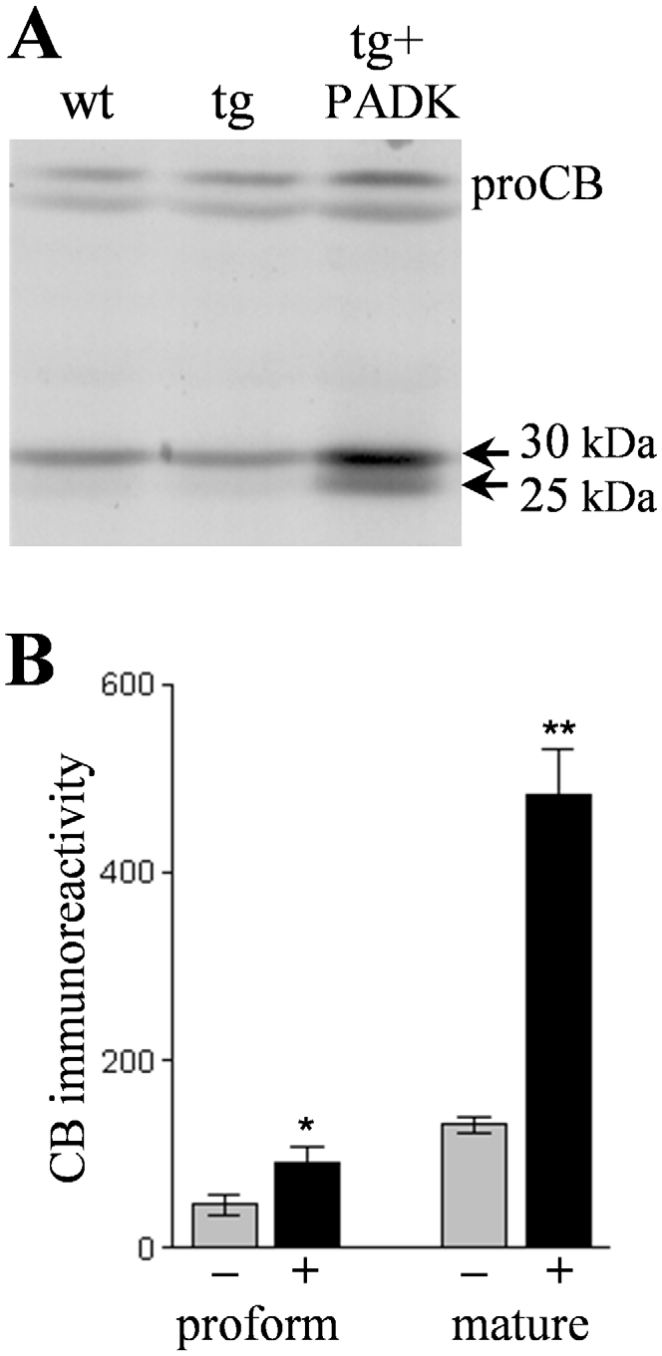
Mature forms of cathepsin B exhibit pronounced modulation by PADK. APP_SwInd_ mice subjected to 9 daily injections of PADK (20 mg/kg; n = 10) or vehicle (n = 9) were assessed for the different cathepsin B species in hippocampal samples. Homogenates from the transgenic mice and from vehicle-treated wildtype mice were analyzed by immunoblot to label the approximately 40-kDa procathepsin B species (proCB) and the 25- and 30-kDa active mature forms (arrows) within the same samples (A). Immunoreactivity levels of the proenzyme and mature forms (means±SEM) in vehicle- (–) and PADK-treated transgenic samples (+) were determined by image analysis (B). Unpaired Mann-Whitney U-test: *P = 0.0296, **P<0.001.

**Figure 5:**
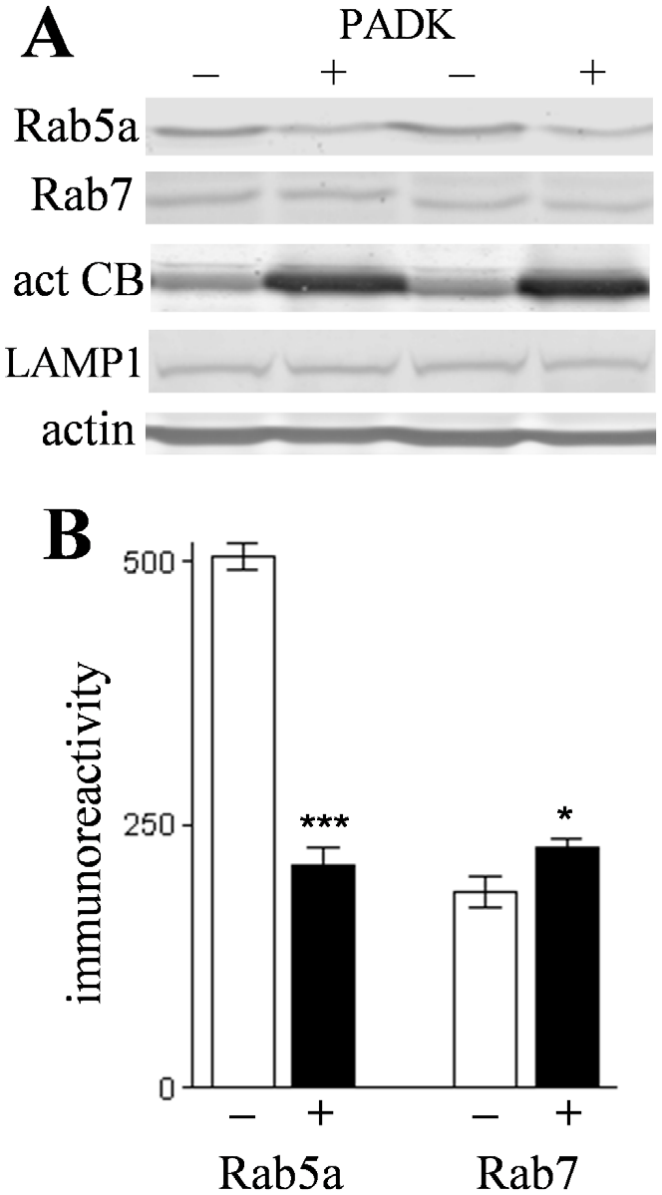
Changes in Rab proteins in cultured hippocampal slices treated with PADK. Stable slice cultures prepared from rat hippocampus were treated daily with PADK (10 µM; n = 12 groups of 8 slices each) or with the corresponding vehicle (final condition of 0.02% DMSO; n = 14 groups of slices) for 4 days. Slice groups were homogenized and equal protein aliquots analyzed by immunoblot for Rab5a, Rab7, active form of cathepsin B (act CB), LAMP1, and actin (A). Rab protein levels (means±SEM) in slices treated without (–) or with PADK (+) were determined by image analysis (B). Unpaired Mann-Whitney U-test: *P = 0.0208, ***P<0.0001.

Hippocampal homogenates from the APP_SwInd_ mice were further analyzed in an attempt to understand how PADK influences lysosomal levels of cathepsin B. Cathepsin B belongs to the superfamily of papain-like cysteine proteases and is first synthesized as a proenzyme. The approximately 40-kDa procathepsin B exhibited a marginally significant increase of 93±27% (mean±SEM; P<0.03) in the PADK-treated group as compared to vehicle-injected transgenic mice samples (Fig. 4A and B). Within the same immunoblot samples, the percent change in the 25–30-kDa mature cathepsin B forms was found to be much larger than the PADK effect on the proform of the enzyme (270±31%, *P*<0.001) (Fig. 4B). PADK's range of effect in the two transgenic models is 4- to 9-fold changes in the mature active isoforms in hippocampus (Table 1), and even greater fold increases when considering the increase in the 25-kDa species alone (see Fig. 4A). These pronounce changes implicate trafficking as a component of the PADK effect, since inactive procathepsin B is processed to the active forms in late endosomes and lysosomes. To selectively examine trafficking markers during defined lysosomal modulation, hippocampal slice cultures were treated with PADK daily for 4 days, causing a >5-fold increase in active cathepsin B. In conjunction with enhanced cathepsin B, the PADK-treated slices exhibited a decrease in Rab5a (Fig. 5A), a marker of early endosomes [34]. From the analysis of integrated optical densities, control slices were found to contain more than 2-fold more Rab5a than the PADK slices, whereas the lysosomal modulator increased the level of Rab7 which controls the late step of endosomal trafficking to lysosomes (Fig. 5B). In addition, as found *in vivo*, LAMP1 was unchanged (control, 156±7.2; PADK-treated slices, 140±5.1; NS). Thus, positive modulation of trafficking likely plays a major role in PADK's ability to increase the content of active cathepsin B in lysosomes and/or late endosomes.

**Figure 6:**
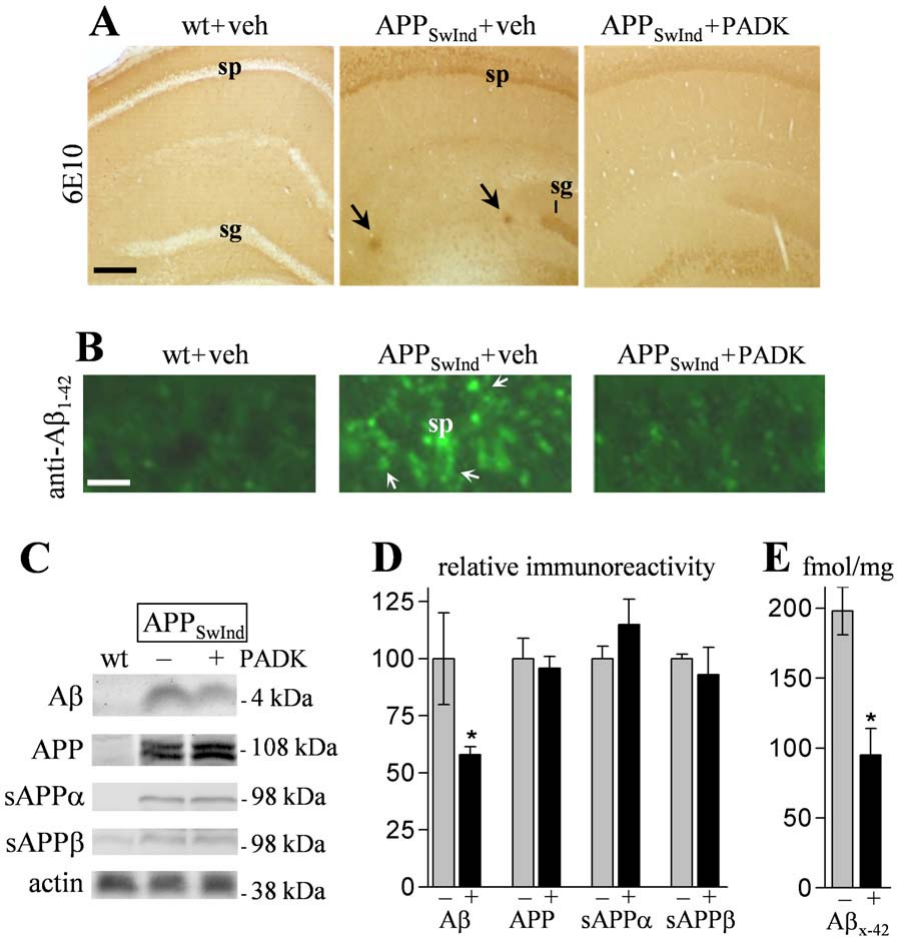
PADK treatment reduces accumulation events in 10–11-month APP_SwInd_ mice. APP_SwInd_ mice were treated with 9 daily injections of either PADK (20 mg/kg; n = 13) or vehicle (veh; n = 10), and wildtype mice (wt; n = 13) were injected with vehicle for 9 days. Fixed brain sections were stained with the 6E10 antibody; arrows denote extracellular deposits (A). Size bar: 450 µm. sg, stratum granulosum; sp, stratum pyramidale. Sections were also immunostained with an Aβ_1–42_–specific monoclonal antibody; arrows denote CA1 cell bodies with intracellular labeling (B; size bar: 15 µm). The remaining tissue was separated for rapid homogenization, and equal protein aliquots of hippocampal samples were assessed by immunoblot. The 6E10 antibody labeled the 4-kDa Aβ species and the parent APP, and selective antibodies were used to label sAPPα, sAPPβ, and actin (C). Positions of molecular weight standards are shown. Mean integrated optical densities±SEM were normalized to 100% in vehicle-treated transgenic samples (–) to allow comparison of PADK effects (+) across the different antigens (D). The samples were also tested with an Aβ_x-42_ sandwich ELISA to determine femtomoles of Aβ_1–42_–related peptides per milligram protein (E). ANOVA: *P*<0.0001; post hoc test compared to APP_SwInd_+vehicle: *P<0.01.

**Figure 7:**
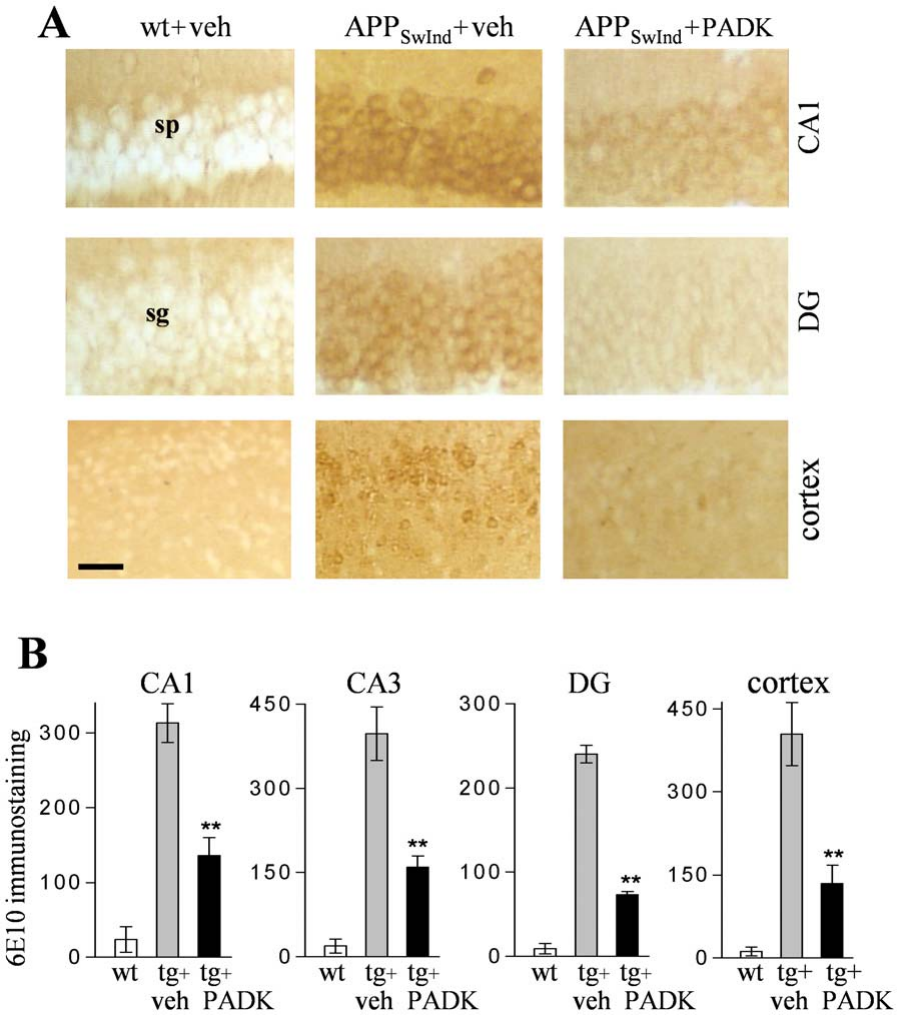
PADK-mediated reductions of intraneuronal accumulation in the APP_SwInd_ mice. Non-transgenic control (wt) and APP_SwInd_ mice were treated with 9 daily injections of PADK or vehicle. Brain sections were stained with the 6E10 antibody, and photomicrographs indicate PADK-mediated reductions of the intracellular labeling in hippocampal subfields and piriform cortex (A). Image analysis for densitometric quantification was conducted across view-fields of four different neuronal layers (B), and mean integrated optical densities were plotted (±SEM). Individual ANOVAs: *P*<0.0001; Tukey's post hoc tests compared to APP_SwInd_+vehicle: **P<0.001. Size bar: 40 µm, CA1 and DG; 65 µm, piriform cortex. DG, dentate gyrus; sg, stratum granulosum; sp, stratum pyramidale.

We next tested whether PADK's influence on the lysosomal system is associated with enhanced Aβ clearance. APP_SwInd_ mice of low transgene copy number were used at 10–11 months of age in order to assess the PADK effects at an early stage of Aβ pathology. Tissue sections were stained with the 6E10 antibody, which specifically recognizes amino acid residues 1–16 of human Aβ. In Figure 6A, intracellular staining is evident among neurons of the stratum pyramidale and stratum granulosum in vehicle-injected APP_SwInd_ mice, while none was found in wildtype mice, and the transgenics also exhibited sporadic extracellular deposits. The daily PADK administration resulted in reduced deposits and a marked decrease in the cellular labeling. Serial sections stained with Aβ_1–42_–specific monoclonal antibody confirmed that Aβ_1–42_ is the primary species that accumulates intracellularly in the APP_SwInd_ brain (Fig. 6B), as previously reported for other AD mouse models [35], and it is the same peptide selectively taken up by neurons of the hippocampus [11]. The Aβ_1–42_ staining intensity in pyramidal neurons was diminished by the PADK treatment, and similar degrees of reduced 6E10 labeling (−63–73%; post hoc tests *P*<0.001) were determined across different neuronal layers in hippocampus and piriform cortex (Fig. 7).

**Figure 8:**
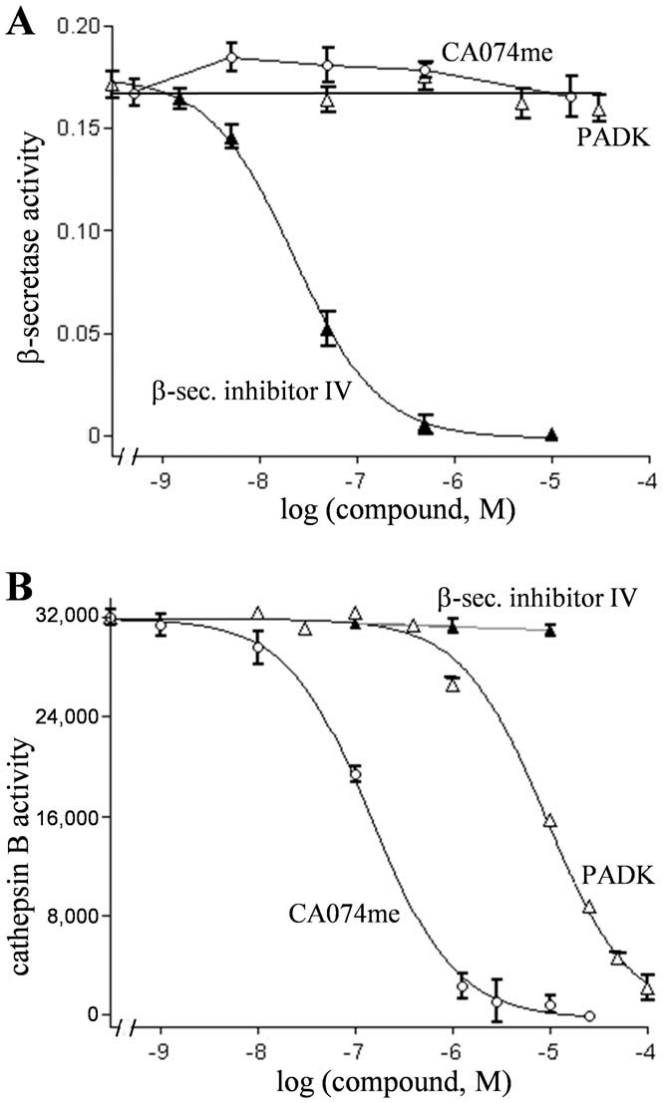
PADK has no inhibitory effect on β-secretase activity. Recombinant human β-secretase (10 ng/ml) was incubated with different concentrations of PADK (open triangles), CA074me (circles), and β-secretase inhibitor IV (closed triangles), and the enzyme activity was determined with the SensiZyme assay kit that uses the procaspase-3 variant containing the β-secretase cleavage sequence Gly-Ser-Ser-Glu-Ile-Ser-Tyr-Glu-Val-Glu-Phe-Arg-Glu-Phe (A). Activity was expressed in absorbance units (mean±SEM), and only β-secretase inhibitor IV elicited inhibition with an IC_50_ of 19.8±2.4 nM. The three compounds were also tested against cathepsin B activity using the fluorogenic substrate Z-Arg-Arg AMC (mean fluorescence units±SEM plotted). β-secretase inhibitor IV had no effect on the cathepsin B activity, and PADK and CA074me resulted in IC_50_ values of 9,200±1,030 and 120±13 nM, respectively (B).

Brain homogenate samples were also analyzed to determine which APP fragments recognized by 6E10 were reduced by the PADK treatment. The lysosomal modulator significantly reduced a 6E10-labeled species of 4 kDa that coincided with the electrophoretic migration of pure Aβ_1–42_ (ANOVA *P*<0.0001, post hoc test *P*<0.01), without affecting the parent hAPP protein level expressed in the transgenic mouse brain (Fig. 6C and D). Similar to the extent of reduction in the 4-kDa peptide, selective measures using the Aβ_x-42_ sandwich ELISA were also significantly decreased by PADK (Fig. 6E), and the decline within each transgenic mouse correlated with the respective level of cathepsin B enhancement in the brain (r = −0.76, *P*<0.05). Other antibodies were used to selectively label sAPPα and sAPPβ which were unchanged after the PADK treatment period, indicating that the APP cleavage activities of α- and β-secretase were not affected (Fig. 6C and 6D). PADK was also found to have no effect on the cleavage activity of recombinant human β-secretase, nor did the potent cathepsin B inhibitor CA074me (Fig. 8).

**Figure 9:**
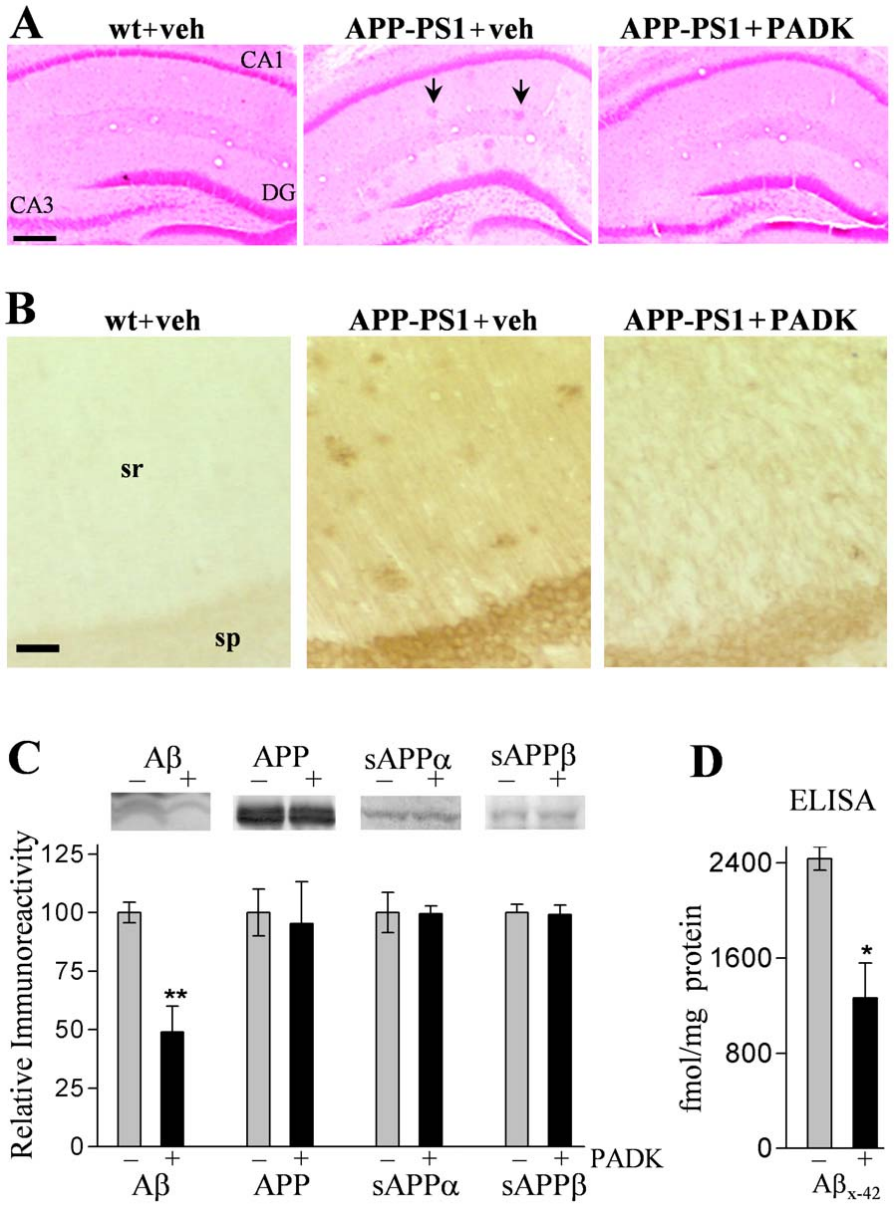
PADK decreases intra- and extracellular 6E10 staining in APPswe/PS1ΔE9 (APP-PS1) mice of 20–22 months. The APP-PS1 mice received 11 daily injections of PADK (i.p., 20 mg/kg; n = 11) or vehicle (veh; n = 10), and non-transgenic control mice (wt) received vehicle injections. Fixed brain sections from the different groups were hematoxylin-eosin stained (A; arrows denote typical deposits) and 6E10 immunolabeled (B), indicating that PADK treatment reduces intra- and extracellular deposition in hippocampus. Equal protein samples from vehicle- (–) and PADK-treated (+) APP-PS1 mouse brains were immunoblotted with 6E10 antibody to assess the 4-kDa Aβ peptide and the parent hAPP, and with selective antibodies to label sAPPα and sAPPβ (C). Mean integrated optical densities±SEM for the different species were normalized to 100% as shown. The same brain samples were also tested by Aβ_x-42_ sandwich ELISA to determine femtomoles of peptide per milligram protein (D). ANOVA: *P*<0.0001; post hoc test compared to APP-PS1+vehicle: **P<0.001. Unpaired t-test: *P<0.01. Size bar: 400 µm, A; 50 µm, B. DG, dentate gyrus; sp, stratum pyramidale; sr, stratum radiatum.

To assess the PADK effects at a more extensive stage of Aβ pathology, we utilized double transgenic APP-PS1 mice of 20–22 months. Their extracellular deposits of 50–90 µm were easily detected in hippocampus and other brain regions in hematoxylin-eosin stained sections (see Fig. 9A) as well as with 6E10 immunolabeling (Fig. 9B). The 6E10 antibody produced very faint background staining in wildtype control mice, whereas pronounced intra- and extracellular labeling was evident in brain sections of the vehicle-treated transgenics. As in the younger APP_SwInd_ mice, PADK-mediated lysosomal modulation in APP-PS1 mice (20 mg/kg/day×11 days, i.p.) was associated with smaller and fewer extracellular deposits as well as reduced levels of intraneuronal staining (Fig. 9A and B). The specific immunostaining intensity was decreased 80% in the CA1 pyramidal layer, and in close correspondence were 76–85% reductions in the above-threshold staining area for extracellular deposits in the stratum radiatum and piriform cortex (ANOVA: *P*<0.0001, post hoc test *P*<0.001; n = 11) (Table 3).

**Table 3:** PADK decreases 6E10 immunostaining in APP-PS1 mouse brain.

	wt	APP−PS1+veh	APP−PS1+PADK
hippocampal sp (IOD)	129±15.1	672±58.9	241±15.0**
hippocampal sr (area)	0.07±0.02	2.90±0.71	0.49±0.08**
piriform cortex (area)	0.13±0.03	3.89±0.36	1.04±0.21**

APP-PS1 mice were injected i.p. daily with PADK (20 mg/kg; n = 11) or vehicle (n = 10) for 11 days. Fixed tissue was sectioned and stained with the 6E10 antibody. Image analysis for densitometric quantification of the immunostaining (mean integrated optical density±SEM) was conducted across view-fields of the hippocampal CA1 stratum pyramidale (sp). Area of deposit labeling above background was also measured for view-fields of the hippocampal stratum radiatum (sr) and piriform cortex (mean percent of total measured area±SEM). ANOVAs: *P*<0.0001; Tukey's post hoc tests compared to APP−PS1+vehicle.

**P<0.001.

**Figure 10:**
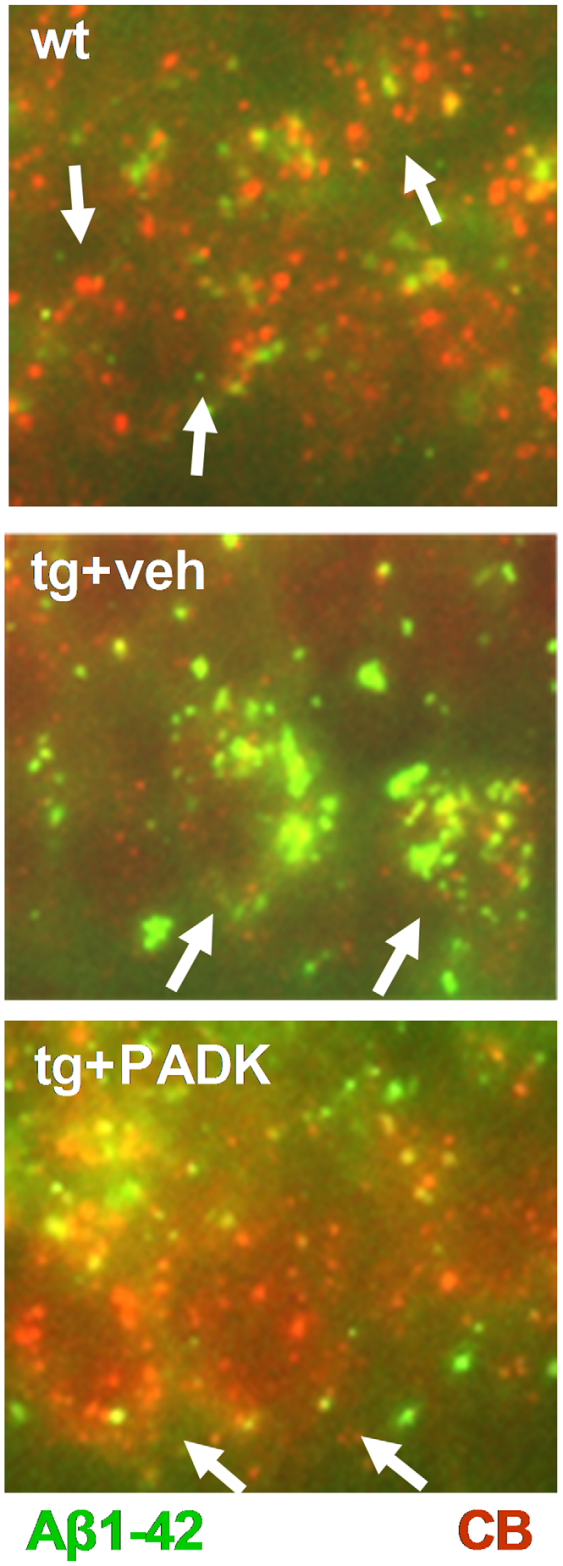
Reduced intracellular Aβ_1–42_ staining corresponds with enhanced cathepsin B. Fixed brain sections from vehicle-treated wildtype mice (wt) and from the APP-PS1 mice treated with vehicle (veh) or PADK were double-stained for Aβ_1–42_ (green) and cathepsin B (red). Immunofluorescence images of CA1 pyramidal neurons (arrows) are shown, with view-field widths of 56 µm.

Brains from the PADK-injected APP-PS1 mice also exhibited a reduction in the 4-kDa Aβ band labeled in 6E10 immunoblots (ANOVA *P*<0.0001, post hoc test *P*<0.001), whereas no decrease was evident in the parent APP (Fig. 9C). The lysosomal modulator similarly reduced Aβ species assessed in immunoblot samples of extracellular-enriched brain extracts, without affecting levels of full-length or secreted forms of APP. As conducted in the APP_SwInd_ model to determine which Aβ species is influenced by PADK, vehicle- and PADK-treated APP-PS1 samples were also subjected to a sandwich ELISA to selectively assess Aβ_x-42_ species (Fig. 9D). The measured concentrations of peptide (femtomoles per milligram homogenate protein) indicate that PADK reduced Aβ_x-42_ by 44–62% (*P*<0.01). This level of reduction closely matches the PADK-mediated 40–63% decrease in the 4-kDa peptide labeled by 6E10, and the peptide is the only antigen recognized by 6E10 that exhibits a reduction in PADK-treated mice. Brain sections from the three groups of mice were also double-stained for Aβ_1–42_ (green) and cathepsin B (red) to specifically assess intracellular Aβ_1–42_ (Fig. 10). Immunofluorescence images revealed punctate Aβ_1–42_–positive material within CA1 pyramidal neurons, and the intracellular accumulation was reduced by PADK in correspondence with enhanced labeling intensity of cathepsin B-positive organelles. Note that with PADK treatment, co-localization of anti-Aβ_1–42_ and anti-cathepsin B staining occurs in several organelle-like structures.

**Figure 11:**
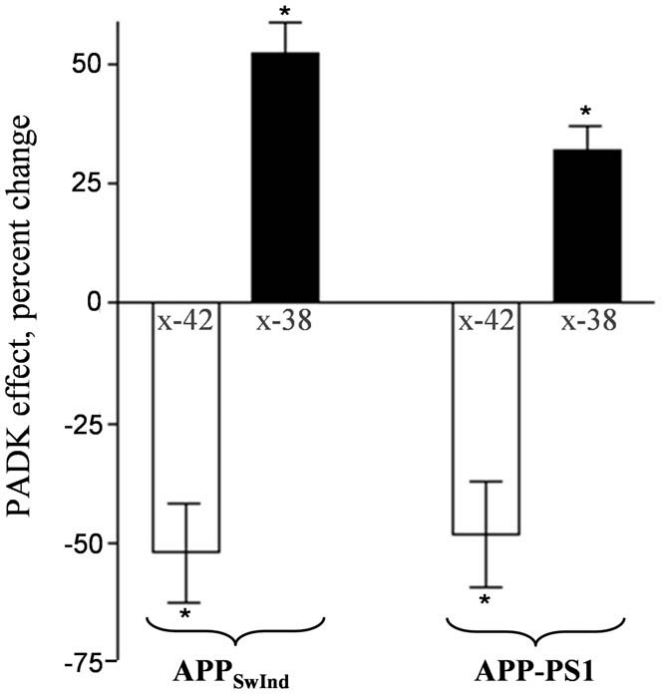
Lysosomal modulator treatment promotes truncation of the Aβ_1-42_ peptide. APP_SwInd_ and APP-PS1 mice treated with vehicle or PADK were assessed for Aβ_x-42_ and truncated Aβ_x-38_ species in brain samples (n = 9−11 per group), using selective sandwich ELISAs. The PADK-mediated changes in Aβ_x-42_ were determined from data presented in Figures 6E and 9D. In the APP_SwInd_ and APP-PS1 samples, PADK increased Aβ_x-38_ species from 60.6±7.7 to 92.2±5.7 fmol/mg and from 104±7.0 to 137±18 fmol/mg, respectively. The plotted PADK effects are expressed as mean percent change±SEM. Post hoc tests compared to the corresponding vehicle-treated transgenic data: *P<0.01.

As evidence points to Aβ_1–42_ being reduced in brains of PADK-treated transgenic mice, the opposite is the case for Aβ_1–38_, a less pathogenic peptide found previously to be a cathepsin B cleavage product of Aβ_1–42_ [16]. Brain samples from APP_SwInd_ and APP-PS1 mice treated with vehicle or PADK were assessed for Aβ_x-42_ and the truncated Aβ_x-38_ species using selective sandwich ELISA protocols (Fig. 11). The Aβ_x-42_ sandwich ELISA does not recognize Aβ_1-38_ or Aβ_1–40_ peptides, and the Aβ_x-38_ ELISA does not recognize Aβ_1–40_ or Aβ_1–42_. The 52% decrease in Aβ_x-42_ measures in PADK-treated APP_SwInd_ mice, as compared to vehicle-treated mice, was matched by the significant 52% increase in Aβ_x-38_ peptide. Regarding the opposing PADK effects in APP-PS1 mice, the 51% decrease in Aβ_x-42_ was partially matched by the significant 32% increase in Aβ_x-38_ species. The transient nature of Aβ_1–38_ production by cathepsin B [16] may account for the smaller change in the truncated peptide. Together with our immunocytochemistry results, Aβ_1–38_ appears to have a lower propensity to resist degradation and accumulate in cells than Aβ_1–42_, as expected from previous studies [11], [17].

**Figure 12:**
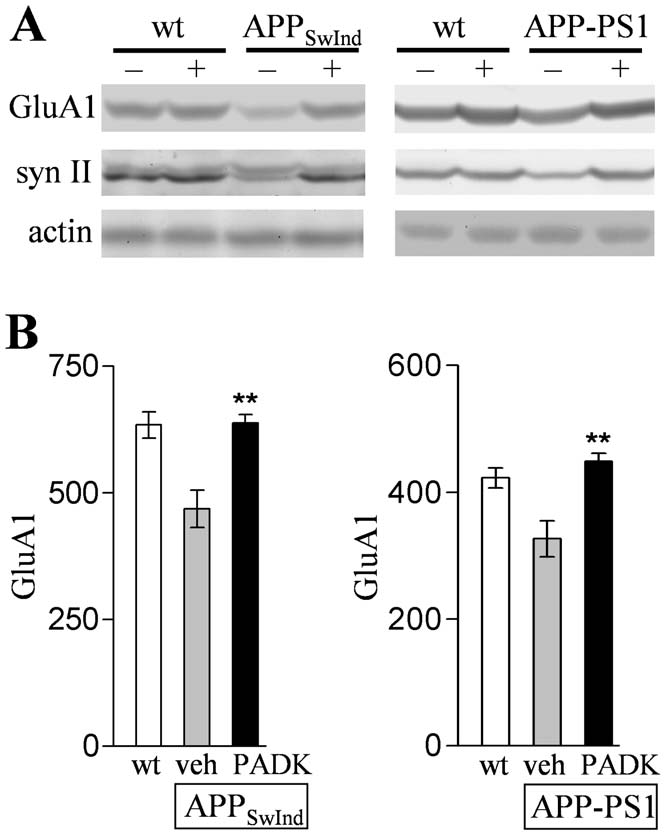
Lysosomal modulation is associated with preservation of synaptic markers in APP_SwInd_ and APP-PS1 mice. Transgenic and wildtype (wt) mice were injected daily with PADK (+) or vehicle (–) for 9–11 days. Equal protein aliquots of hippocampal homogenates were analyzed by immunoblot for synaptic markers and actin, showing PADK-improved levels of GluA1 and synapsin II (syn II) in transgenic mice (A). The mean GluA1 immunoreactivities±SEM are shown for vehicle-treated wildtypes and for the vehicle- and PADK-treated transgenics (B). Post hoc tests compared to vehicle-treated transgenics: **P<0.001.

The lysosomal modulator's effects on Aβ clearance were associated with synaptic protection. In Figure 12A, hippocampal samples from the two mouse models were analyzed by immunoblot for the presynaptic protein synapsin II and the postsynaptic glutamatergic marker GluA1. Similar to the extent of synaptic decline seen in related transgenic mice [30], [33], the synaptic proteins exhibited deficits of 23–31% in APP_SwInd_ and APP-PS1 mice as compared to their respective age-matched wildtypes (*P*<0.01), while actin levels remained unchanged. PADK treatment significantly reduced the GluA1 deficit in the two mouse models, reaching levels comparable to those found in non-transgenic control mice (Fig. 12B) (ANOVA: *P*<0.001; n = 12−20). Similar indications of synaptic protection were found when assessing synapsin II and synaptophysin. The integrity of hippocampal dendritic fields was also found preserved in immunostained tissue sections, and the level of GluA1 immunoreactivity within each transgenic mouse correlated with the respective extent of cathepsin B enhancement in the brain (r = 0.78, *P* = 0.02).

**Figure 13:**
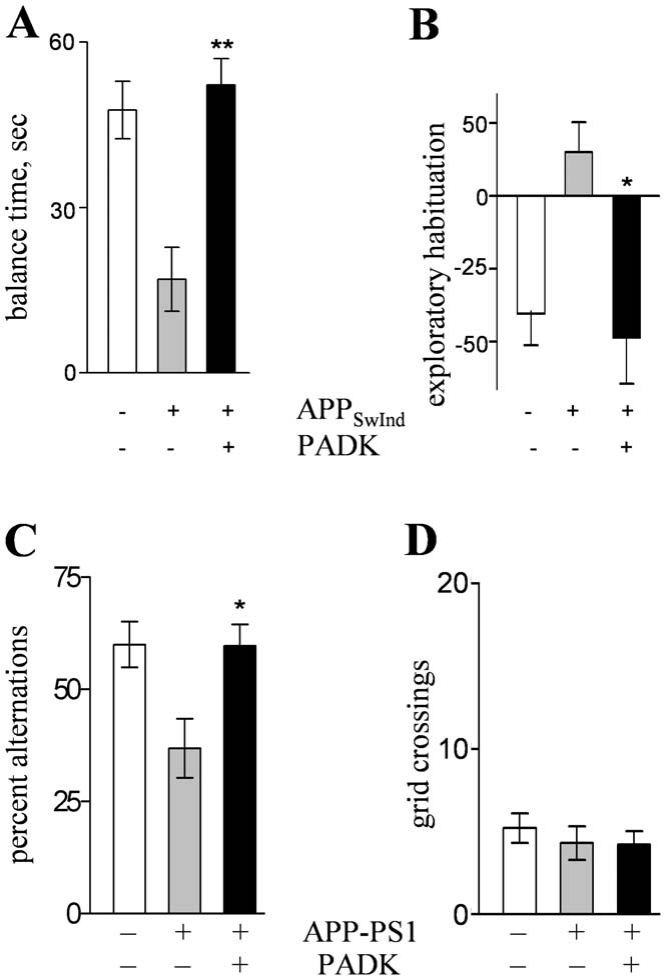
PADK reduces behavioral deficits in APP_SwInd_ and APP-PS1 mice. In the first model, vehicle-treated wildtype mice (n = 11) were tested with groups of vehicle- (n = 10) and PADK-treated APP_SwInd_ mice (n = 13) across trials on the suspended rod test (A), and time maintained on the rod during the third trial was plotted (means±SEM). The animal groups were also tested across consecutive days in the same novel field, and the percent change±SEM in exploratory distance on the second day compared to the first was determined (B). In the second model, age-matched vehicle-treated wildtypes were tested with groups of vehicle- (n = 10) and PADK-treated APP-PS1 mice (n = 11) for spontaneous alternation behavior in a T-maze (C); data are plotted as percent of maximum alternations possible (mean±SEM). Open field mobility assessment confirmed no change in mean grid crossings±SEM across the three groups of mice (D). Post hoc tests compared to vehicle-treated transgenics: *P≤0.01, **P<0.001.

Lastly, we assessed the mouse models to determine whether the PADK-mediated clearance and synaptic protection translate to improvements in behavioral tests. Although the APP_SwInd_ mice used in the present study express low levels of Aβ deposits, their synaptic decline was associated with significant deficits on the suspended rod test (Fig. 13A) as well as on the exploratory habituation test (Fig. 13B), similar to the type of deficit reported previously for APP_SwInd_ mice of the same age range [36]. Compared to wildtypes, the vehicle-injected APP_SwInd_ mice exhibited a marked reduction in balance time while coordinating to reach a safe platform, whereas the PADK-treated transgenics performed at the same level as the control mice (post-hoc test p<0.001) (Fig. 13A). A marked degree of disinhibition was also displayed by the vehicle-treated transgenics on the second day of open field exploration, but APP_SwInd_ mice injected with PADK were completely devoid of this behavioral defect (Fig. 13B). No differences were found among the three groups of mice regarding gross mobility during open field exploration, nor were any differences evident during mobility testing on a slow rotating rod (not shown). Also corresponding with synaptic marker decline, the APP-PS1 mouse model of extensive Aβ pathology expressed cognitive deficiency in the hippocampal-dependent task of spontaneous alternation behavior (Fig. 13C), similar to episodic memory deficits reported previously [33], [37]. The vehicle-treated APP-PS1 mice exhibited reduced alternation behavior of alternate arm entries in the T-maze as compared to wildtype mice (*P*<0.0001). The 11-day PADK treatment resulted in significantly improved alternations, reaching a performance level equal to that of the age-matched wildtypes. Open field assessment confirmed no change in exploratory mobility across the different groups of mice (Fig. 13D). These data confirm in a second AD mouse model that lysosomal modulation reduces behavioral deficits.

## Discussion

Our study indicates that lysosomes are a site of modulation for the targeted enhancement of cathepsin function to promote clearance of the Aβ peptide. The endosomal-lysosomal pathway has been implicated as playing a role in the degradation of Aβ, and the impairment of this pathway likely contributes to the accumulation of intraneuronal Aβ and other proteins linked to AD. Enhancement of the lysosomal system was achieved with the modulatory agent PADK, resulting in the selective increase of cathepsin B activity in lysosomes. The lysosomal modulation led to reductions in both intracellular and extracellular Aβ, to a degree that ameliorates the phenotype of two AD mouse models with distinctly different levels of Aβ pathology. At the analogous stages of the human disorder, reducing Aβ accumulations is thought to be essential for slowing AD progression.

When administered systemically, the PADK modulator increased cathepsin B levels 4- to 8-fold across different brain regions in APP_SwInd_ and APP-PS1 mice. From the analysis of immunofluorescence images, enhanced intracellular cathepsin B staining was found to represent the primary PADK effect in neurons. As cathepsin B increased in the transgenic mice, there was a corresponding decrease in Aβ_1–42_ measured by selective ELISA. Brain immunocytochemistry with anti-Aβ_1–42_ and 6E10 antibodies suggests that lysosomal modulation leads to decreased levels of intraneuronal deposition. Interestingly, reduction in extracellular deposits occurred in correspondence with the reduced labeling of intracellular Aβ, providing evidence of an equilibrium relationship between secreted Aβ peptides and the extracellularly deposited material. This fits well with Oddo et al. [35] which described the accumulation of intracellular Aβ as a source of extracellular amyloid deposits. Note that PADK had no effect on hAPP expression levels in the transgenic brains, whereas the 4-kDa Aβ species was markedly reduced. This 44–63% decrease in Aβ likely contributes to the reduced extracellular deposition as multiphoton microscopy and microdialysis studies showed that reduction of Aβ by as little as 20–25% was capable of diminishing plaque deposits [38]. The actions of PADK on cathepsin B and Aβ indicate the intraneuronal clearance process as an appropriate target for offsetting protein accumulation pathology. Since extracellular peptide taken up by vulnerable neurons is thought to contribute significantly to the accumulation of intracellular Aβ_1–42_ [11], [17], [18], [39], the modulated lysosomal system may be exerting its clearance effects directly on the internalized Aβ_1–42_ peptide as well as by increasing the turnover of Aβ-containing APP fragments. Lysosomal modulation in other cell types will be the subject of follow-up studies since in microglia, internalized oligomeric Aβ has recently been shown to be trafficked to lysosomes and degraded by cysteine proteases including cathepsin B [40].

PADK-mediated lysosomal modulation appears to involve both increased expression of cathepsin B and enhanced trafficking of the enzyme. The modulation consisted of a modest increase in procathepsin B as compared to larger 4- to 9-fold increases in the mature active forms of the enzyme. Further evidence that PADK has a positive effect on endo-lysosomal trafficking includes i) cathepsin B hydrolytic activity was enhanced in isolated lysosomes to a much greater degree than the enhancement of proenzyme in brain samples, ii) active cathepsin B forms were enhanced in the absence of any increase in the lysosomal marker LAMP1, iii) the modulated cathepsin B co-localized with LAMP1 in neurons, and iv) decreases in the Rab5a early endosome marker corresponded with the enhanced levels of mature cathepsin B in hippocampal tissue. Elevated expression of early endosome regulators, on the other hand, occur in hippocampal neurons from individuals with AD and mild cognitive impairment, suggesting that early endosomal dysfunction contributes to AD progression [27], [41]. The action of PADK may offset endocytic dysfunction and promote protein clearance through enhanced trafficking of cathepsin B and/or Aβ-containing species from early endosomes to late endosomes and lysosomes. Perhaps enhanced cathepsin B maturation is facilitated by the small PADK-mediated increase in mature cathepsin D, since previous studies found the latter to activate procathepsin B into mature forms [42], [43]. Regarding PADK's lack of influence on LAMP1, this would appear to rule out any broad effect on lysosome production or the modulation of TFEB, a transcription factor that regulates lysosomal biogenesis [44], [45]. TFEB does share with PADK the ability to elicit protection in models of pathogenic accumulation events, as shown in TFEB-transfected cells insulted with a parkinsonian neurotoxin [45].

An obvious paradox follows from PADK promoting lysosomal function while historically being classified as an inhibitor of the thiol proteases cathepsins B and L. Indeed, extended exposure of hippocampal slice cultures to >40 µM PADK causes multiple signs of lysosomal dysfunction and the characteristic proliferation of a lysosomal marker [46], [47]. On the other hand, low concentrations of 1–10 µM significantly increased cathepsin enzymes without producing lysosomal pathology or synaptic decline over extended treatments of 5–20 days [19], [20], [46], and without any obvious axonopathy, swelling of axonal initial segment, or transport failure. In fact, the low-level PADK treatment led to enhanced clearance in the hippocampal slice model of protein accumulation pathology, as well as to recovered levels of microtubule stability markers, transport function, tubulin-binding proteins, and pre- and postsynaptic proteins. Here, comparable effects on Aβ clearance and synaptic protection were produced by PADK in transgenic mice.

The pronounced up-regulation of cathepsin B and the changes in Rab proteins are not the first paradoxical effects found produced by a cathepsin inhibitor. PADK causes increased proenzyme expression in the transgenic mouse brains, likely related to the paradoxical findings in which the protease inhibitor leupeptin increased cathepsin activities in different tissues. The broad-acting inhibitor, as high as 200 mg/kg, caused significant inhibition of cathepsins B and L, but after clearance of the administered leupeptin not only did cathepsin activities recover to control levels they continued to increase more than two-fold [48]–[51]. Note that high levels of PADK administered to mice (60 mg/kg i.p. in 100% DMSO) or to 3T3 fibroblasts (100 µM applied daily) found no such increases in cathepsin B and L activities [48], [51]. However, the study that tested lower PADK concentrations reported increased amounts of the 39-kDa procathepsin L [51], perhaps related to the increase in procathepsin B in the present study. Unique compared to leupeptin, PADK had a long-term effect on increasing the active intermediate of a cathepsin. In fact, they showed that their lowest concentration tested, 3 µM, led to pronounced accumulation of the active 29-kDa lysosomal form. The buildup of cathepsin intermediates may signify modulated enzyme trafficking, resulting in a larger pool of accessible isoforms for more efficient maturation, and thereby improving lysosomal efficiency.

The enhanced lysosomal capacity described here is through targeted enzyme modulation, producing selective enhancement of a clearance pathway since PADK was found to increase cathepsin B activity whereas neprilysin and insulin-degrading enzyme levels were unchanged in the APP_SwInd_ and AP-PS1 mice. In the normal brain, cathepsin B may act together with identified Aβ-degrading enzymes to provide efficient Aβ clearance. Extracellular and, in some cases, intracellular Aβ levels have been shown to be decreased by neprilysin, insulin-degrading enzyme, and endothelin-converting enzyme [12]–[15]. Note that correlational analyses found a negative correlation between neprilysin expression and Aβ accumulation as well as clinical diagnosis, but this was not the case regarding the expression of other Aβ-degrading enzymes [52]. Lysosomal integrity and cathepsin B regulatory responses are perturbed in the aged brain [16], [53], suggesting that age-related disruptions of effective cathepsin B and neprilysin activities together contribute to the aging risk factor of AD.

The lack of change regarding sAPPα and sAPPβ in both transgenic models also rules out PADK's modulation of α- or β-secretases. Positive modulation of β-secretase would promote Aβ production in contrast to the reduction of Aβ species found elicited by PADK. PADK was absent of any inhibitory action on β-secretase activity as well, thus ruling out the weak cathepsin inhibitor's ability to influence Aβ levels by blocking β-site cleavage of APP. Positive regulation of α-secretase has the potential to preclude Aβ production. The α-secretase enzyme has also been implicated in an alternative route of clearance in which cathepsin-mediated elevation of α-secretase activity regulates Aβ production [54], [55]. PADK's influence on cathepsins may indirectly utilize α-secretase, however, PADK effects did not include altered α-secretase or sAPPα levels.

A defect in proteolytic mechanisms that can degrade Aβ or its precursors may constitute a major determinant of AD pathogenesis and progression, especially in late-onset sporadic AD [56]. Perhaps related to this, reduced efficiency of the lysosomal system leads to the buildup of amyloidogenic species [46], [57]–[59] and other proteins linked to aggregation events and synaptotoxicity [19], [22], [60]–[62]. Of particular interest in regards to the present study, genetic inactivation of cathepsin B resulted in increased abundance of Aβ_1–42_ and the worsening of Aβ pathology [16]. Correspondingly, elevating cathepsin B expression or net cathepsin activity leads to protection against Aβ pathology [16], [59], and purified cathepsin B was found to cleave Aβ_1–42_ into shorter less pathogenic peptides [16]. Here, the induced increase in cathepsin B activity in lysosomes points to this enzyme's modulation as being involved in PADK's protective clearance of Aβ_1–42_. In fact, increases in the active form of cathepsin B correlated with the degree of Aβ_1–42_ reduction, and co-localization of Aβ_1–42_ and cathepsin B occurred in lysosome structures. In contrast to the reduced levels of Aβ_1–42_ peptide, PADK-treated transgenic mice exhibited an increase in the shorter Aβ_1–38_ species, indicating that the protective modulation involves the truncation of Aβ_1–42_. The corresponding increase in the Aβ_1–38_ peptide as Aβ_1–42_ was reduced supports the direct intracellular processing by modulated lysosomes as a route of Aβ detoxification.

Reducing the accumulation of Aβ peptides, especially the aggregation-prone species Aβ_1–42_, is the main objective of many approaches attempting to reduce synaptopathogenesis and associated cognitive defects in AD. Aβ species are known to disrupt neurotransmission, synaptic plasticity, and memory encoding [6], [31], [63]–[67], and cause the loss of synaptic integrity [2], [4], [8], [11], [68], [69]. Loss of synaptic markers was evident even at early stage Aβ pathology in the 10–11-month APP_SwInd_ mice, and APP-PS1 mice exhibited distinct declines in pre- and postsynaptic proteins as well. PADK-mediated lysosomal modulation and Aβ clearance translated into restoration of synaptic components, perhaps by reducing specific accumulation events that have been implicated in transport failure and axonopathy. Associated with the synaptic decline, APP_SwInd_ mice displayed balance and coordination defects and failed to recognize a familiar environment, and the older APP-PS1 mice exhibited deficits in hippocampal-dependent spontaneous alternation behavior. These data provide evidence of intracellular Aβ_1–42_ accumulation correlating with functional compromise. Corresponding with the synaptic protection, the lysosomal modulator treatment significantly improved performance in both mouse models, in which behavioral measures in PADK-treated transgenics were comparable to those found in non-transgenic control mice.

The opposing effects of lysosomal modulation on cathepsin B vs. Aβ_1–42_ levels support the idea that the lysosomal pathway works, at least in part, towards the clearing of toxic Aβ peptides. The lysosomal system responses to a pharmacologically plausible enhancement strategy that is mechanism based, resulting in intracellular Aβ clearance that also leads to reduction of extracellular deposits. These findings support new ideas regarding Aβ metabolism and the equilibrium events that influence extracellular deposition. Future work has the potential to identify lysosomal modulatory agents that have specific actions to best alleviate protein accumulation pathology. For instance, modulators of lysosomal biogenesis could tap into the TFEB pathway, thereby eliciting broad action across several lysosomal enzyme systems. Decoupling the inhibitory nature of PADK from its trafficking modulation effects may provide an important direction to develop agents that specifically enhance cathepsin maturation for promoting protein clearance. PADK's weak inhibitory potency may explain its effect on procathepsin B as in the case of previously reported leupeptin effects, whereas the influence on mature cathepsin forms is most apparent with PADK treatment. The corresponding amelioration of synaptic and behavioral deficits in transgenic models suggests that lysosomal enhancement can provide effective protection at different stages of AD pathogenesis, thus having important implications for the development of disease-modifying therapies.
